# Macular hole repair using a refined viscoelastic assisted membrane positioning technique markedly improves surgical success rates

**DOI:** 10.1007/s10792-025-03466-w

**Published:** 2025-03-14

**Authors:** Anny M. S. Cheng, Shailesh K. Gupta, Nizar S. Abdelfattah, Kakarla V. Chalam

**Affiliations:** 1 Department of Ophthalmology, Broward Health, Fort Lauderdale, FL USA; 2 Specialty Retina Center, Pompano Beach, FL USA; 3 Department of Ophthalmology, Florida International University, Herbert Wertheim College of Medicine, , Miami, FL USA; 4 Department of Ophthalmology, Loma Linda University, 11370 Anderson St., Suite 1800, Loma Linda, CA 92354 USA

**Keywords:** Epiretinal membrane, Inverted internal limiting membrane flap, Macular hole, Viscoelastic-assisted, Internal limiting membrane

## Abstract

**Introduction:**

To evaluate the surgical outcomes of pars plana vitrectomy (PPV) using dispersive viscoelastic-assisted inverted internal limiting membrane (VILM) flap technique in patients with full-thickness macular holes (MH).

**Methods:**

We retrospectively review 247 eyes that underwent PPV with the VILM flap technique and had a minimum follow-up of six months. Following core vitrectomy, the internal limiting membrane (ILM), stained with indocyanine green, was placed on the MH. A dispersive viscosurgical device was applied over the ILM flat to secure its placement before fluid-gas exchange. Sulfur hexafluoride gas was used for tamponade, and patients maintained face-down positioning postoperatively. The primary outcomes assessed were anatomical closure of MH at 90 days confirmed with optical coherence tomography and changes in best-corrected visual acuity (BCVA).

**Results:**

Anatomical closure of MH was achieved in 98% of eyes at 90 days postoperatively. Success rates were inversely correlated with MH diameter: 100% for small (< 400 μm; n = 122), 98% for medium (400–800 μm; n = 102), and 91.3% for large (> 800 μm; n = 23) holes. The median improvement in BCVA was 0.3 logMAR. Significant improvement in BCVA was observed at three months (p = 0.025) and six months (*p* = 0.019) postoperatively. Final BCVA improved in 87% of eyes (n = 215), remained stable in 11% (n = 27), and worsened in 2% (n = 5). No cases of ILM flap displacement were noted.

**Conclusion:**

The VILM flap technique during PPV is highly effective for treating MH, demonstrating high anatomical closure rates and significant visual improvement. This method enhances the likelihood of successful outcomes after initial surgery.

**Supplementary Information:**

The online version contains supplementary material available at 10.1007/s10792-025-03466-w.

## Introduction

Macular hole (MH) is a common cause of poor visual acuity in the elderly population, and traditional surgery involves vitrectomy with gas tamponade [1]. Vitrectomy has been the gold standard of MH treatment, and traditionally, internal limiting membrane (ILM) peeling is performed to relieve the anteroposterior and tangential traction on the retinal surface, with studies reporting closure rates for MH ranging from 94 to 98% [2, 3].

However, the closure rate for large macular holes is variable. Large MHs are associated with an increased risk of failure, and the closure rate with the ILM peeling technique ranges from 40 to 80% [4–7]. Recently, several methods have been developed to enhance the closure rates of MHs. In 2010, Michalewska et al. [8] first published the inverted ILM flap technique, which demonstrated a high success rate in closing MHs. This approach involves leaving the ILM intact to cover the MH, thereby aiding in the closure of the hole and promoting the regeneration of the retinal structure. Nevertheless, the original approach had drawbacks, such as the occurrence of spontaneous retroversion of the ILM flap in approximately 14–20% of patients during the fluid-air exchange, which can lead to initial failure. Additionally, a coiled portion of the peeled ILM is employed to fill the MH in a manner resembling a plug, which can deviate from the natural and typical foveal shape and may result in excessive gliosis.

Subsequently, Shin et al. [9] introduced a flap technique with Perfluoro-n-octane (PFO) assistance; however, flap dislocation during fluid-air exchange remained an issue, resulting in an 83% hole closure rate. In 2015, Michalewska modified his technique to a temporal inverted ILM flap [10], achieving better outcomes regarding flap displacement and hole closure, with only 2 out of 87 eyes experiencing flap displacement. In 2018, Chen introduced a free-flap technique using viscoelastic agents to tamponade the flap [11]. Visco-assisted surgery, which includes the insertion of multiple free ILM flaps, resulted in a 100% closure rate for MH in 13 eyes [12].

In our technique, we combine the latter two methods by employing a hinged inverted ILM flap with dispersive viscoelastic material placed over the flap to secure it in place and minimize risk of dislocation during fluid-gas exchange. This report represents one of the most extensive analyses of MH closure rates utilizing a dispersive viscoelastic-assisted ILM (VILM) flap method.

## Methods

The inclusion criteria were clinical diagnosis of MH (ICD-9 code: 362.54) without concurrent retinal or choroidal detachment, and a minimum diameter of the MH of 100 μm. The exclusion criteria included a history of retinal detachment or proliferative vitreoretinopathy, any type of retinal surgery, diabetic retinopathy, vitreous hemorrhage, retinal vascular occlusion, uveitis, trauma, optic atrophy, ocular tumors, glaucoma, corneal opacity, or incomplete chart records.

**Fig. 1:**
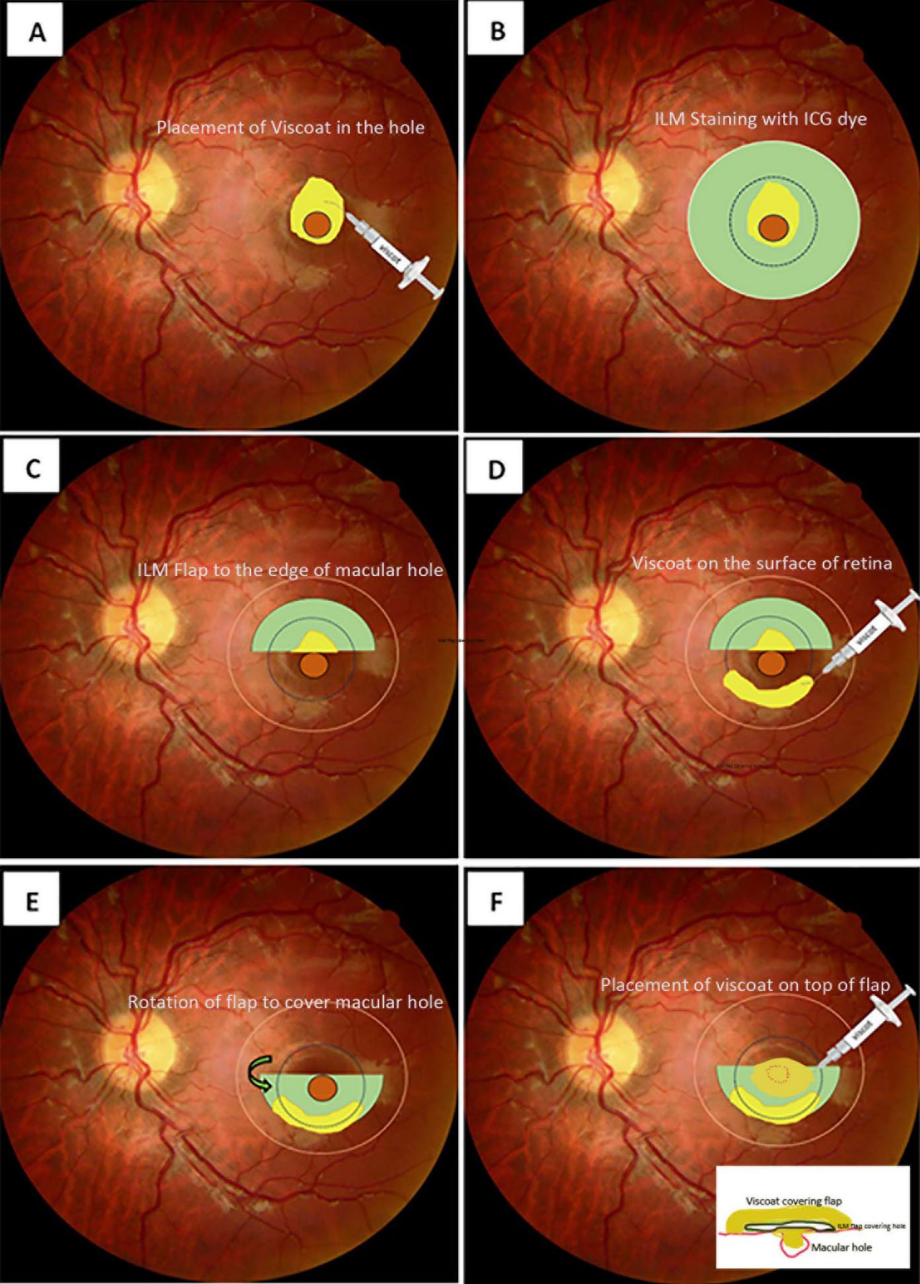
Surgical steps to implement the inverted internal limiting membrane (ILM) flap technique. **A** Placement of Viscoat into the macular hole (MH). **B** Indocyanine Green (ICG) was placed around the MH to stain ILM. **C** The ILM was removed in a circular manner. The superior ILM remained connected to the superior edge of the MH, creating an ILM flap of approximately one disk diameter. **D** Viscoat as an adhesive was injected around the MH in the bottom half of the macular region. **E** The ILM flap was then flipped inferiorly to cover the whole MH. **F** An additional layer of Viscoat was placed as a weight on top of the inverted ILM flap, which covered the MH

All eyes involved in this research underwent the standard 23-gauge 3-port pars plana vitrectomy (PPV) procedure under retrobulbar anesthesia. Vitrectomy was conducted using the Bausch&Lomb: MillenniumTM vitrectomy system (Rochester, NY, USA). The 23-gauge instrument platform provides significantly stiffer instruments compared with 25- or 27-gauge systems, which allow for improved intraoperative eye stability and better positioning of the ILM flap. A posterior vitreous detachment was initially induced, followed by the extraction of the remaining fine premacular posterior cortex. If an epiretinal membrane was present, it was removed using forceps. A dispersive ophthalmic viscosurgical device (VISCOAT, Alcon Laboratories, Fort Worth, TX, USA) was administered into the MH and the corresponding symmetrical region above the MH (Fig. 1A). A solution of Indocyanine Green (ICG) 0.125% was meticulously applied around the MH located within the arcades (Fig. 1B). Excess ICG and VISCOAT were then removed using suction. Subsequently, the ILM was carefully raised in a circular manner, covering an area approximately 2.5 times the diameter of the optic disc surrounding the MH. The superior ILM was not fully detached from the retina; instead, it remained connected to the superior edge of the MH, creating an ILM flap of approximately one disk diameter (Fig. 1C). VISCOAT was injected and distributed in an arched shape around the MH in the lower half of the macular region to serve as an adhesive (Fig. 1D). The ILM flap was then flipped inferiorly by turning it inside out with intraocular forceps to cover the entire MH (Fig. 1E). It was subsequently gently massaged to flatten it. Additional VISCOAT was applied as a weight on top of the inverted ILM flap (Fig. 1F).

At the end of the procedure, an fluid-gas exchange was performed using a 25% concentration of sulfur hexafluoride (SF6) as a tamponade (Supplementary Video). Subsequently, the patients were maintained in a prone position (facedown) for a duration of three days. Each patient underwent a comprehensive ophthalmic evaluation, which included refraction and measurement of best-corrected visual acuity (BCVA), examination with a slit-lamp, indirect ophthalmoscopy, and spectral domain optical coherence tomography (SDOCT, Heidelberg Engineering GmbH, Heidelberg, Germany). These evaluations were conducted at baseline preoperatively and at 1 week, 1 month, 3 months, and 6 months postoperatively. The BCVA was converted into the logarithm of the minimum angle of resolution (log MAR) for the purposes of statistical analysis.

Statistical analysis was performed with GraphPad Prism version 10.0.0 for Windows, GraphPad Software, Boston, Massachusetts USA (www.graphpad.com). Descriptive statistics were employed to summarize patient demographics and clinical information in terms of means and standard deviations. The BCVAs (log MAR values) before and after surgery were assessed using the paired t-test. Correlations between parameters were analyzed using the Pearson correlation coefficient. A p-value of less than 0.05 was considered statistically significant.

## Results

**Fig. 2:**
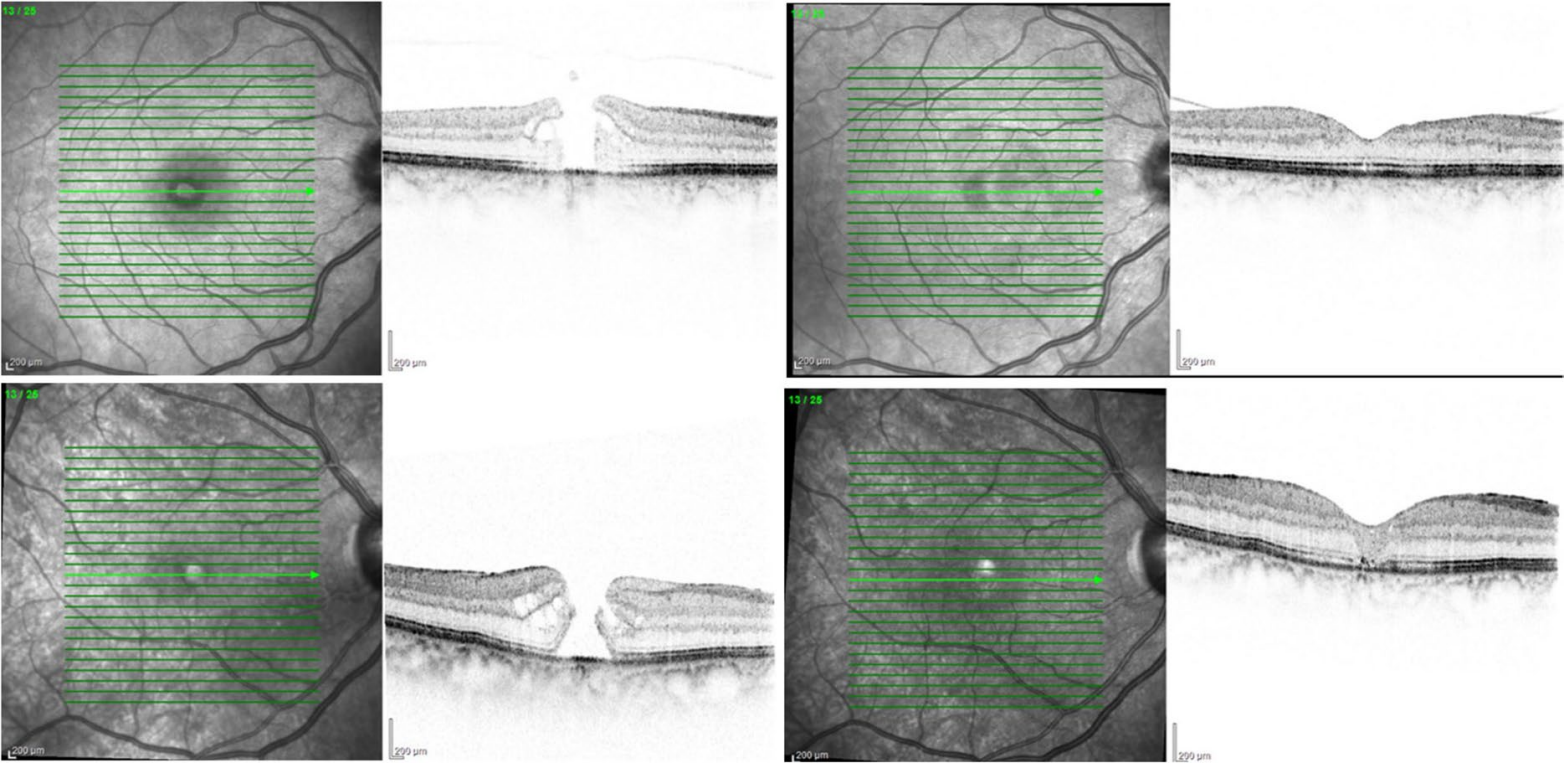
Example cases from study patients. The upper row, patient 1, displays preoperative baseline OCT (left) in comparison to postoperative OCT at the 3-month follow-up (right). The lower row, patient 2, presents preoperative OCT (left) juxtaposed with postoperative OCT at the 6-month follow-up (right)

Table 1 presents the fundamental clinical features of all patients. The average duration of follow-up was 8.7 ± 2.0 months, with a range of 6.0 to 11.2 months. No significant intraoperative complications occurred in any of the cases. There were no instances of the inverted ILM flap reversing during the fluid-gas exchange. A total of 243 eyes (98%) achieved complete closure of the MH following the first PPV using the VILM flap method (Fig. 2). Of the 243 eyes analyzed, 225 eyes (91%) demonstrated type 1 closure (MH closed without any deficiency in the foveal neurosensory retina). The remaining 18 eyes (7.3%) exhibited type 2 closure (MH closed but with a foveal neurosensory retinal defect).

**Table 1:** Preoperative baseline clinical features

Sample Size (eyes)	247
Age (years) Mean ± Standard Deviation Range	50.1 ± 8.8 21–70
Gender Male Female	132 115
Race Caucasian Asian African American	222 12 13
Lens Status Phakic Pseudophakic	158 89
MH Diameter (micrometer) < 400 (small) 400–800 (moderate) > 800 (large)	122 102 23
Duration of Follow- up (months) Mean Range	8.7 ± 2 6.0–11.2

The average preoperative BCVA was 1.18 ± 0.56 logMAR. The mean BCVA at 1 week, 1 month, 3 months, and 6 months postoperatively was 1.21 ± 0.96, 1.09 ± 0.64, 1.10 ± 0.75, and 1.08 ± 0.68, respectively. Overall, there was a notable improvement in BCVA at 3 months (*p* = 0.025) and 6 months (*p* = 0.019) postoperatively when compared to the preoperative baseline (Table 2). However, no significant improvement was observed at 1 week and 1 month postoperatively. At the last follow-up examination, BCVA improved in 215 eyes (87%), remained stable in 27 eyes (11%), and worsened in 5 eyes (2%). The median improvement in BCVA was 0.3 logMAR. The improvement in BCVA correlated with the visual acuity at baseline (r = 0.76, *p* = 0.03) but did not correlate with the size of MH at presentation (*p* = 0.7).

**Table 2:** Macular features and postoperative outcomes

Variable	Number (%)	*P value*
MH Diameter (micrometer) Pre-op	527.60 ± 105.8	–
Complete Closure in different MH Diameters < 400 (small) 400–800 (moderate) > 800 (large)	242 (98%) 122/122 (100%) 100/102 (98%) 21/23 (91.3%)	–
Type 1 (closed without foveal neurosensory retinal defect)	224 (90.6%)	–
Type 2 (closed with foveal neurosensory retinal defect)	18 (7.3%)	–
Change in BCVA Improved Unchanged Worsened	215 (87%) 27 (11%) 5 (2%)	–
Visual Acuity (log MAR) Baseline 1 week 1 month 3 months 6 months	1.18 ± 0.56 1.21 ± 0.96 1.09 ± 0.64 1.10 ± 0.75 1.08 ± 0.68	0.2 0.1 0.025 0.019

## Discussion

The effectiveness of the inverted ILM flap method is crucial in the treatment of macular hole (MH), particularly those larger than 400 microns, as larger MHs present a more challenging surgical task. Large MHs are more likely to experience failure of closure or reopening after initially being successfully closed, compared to small MHs [13]. A recent meta-analysis has indicated that the inverted ILM flap technique may offer benefits over regular ILM peeling, especially in terms of achieving higher closure rates (RR 1.10, 95% CI 1.02 to 1.18; *p* = 0.01) for large MH [14]. The challenge in this approach lies in ensuring that the inverted ILM flap remains in place while performing the subsequent fluid-gas exchange manipulation.

According to reports, 14% of cases experience spontaneous retroversion of the free end of the ILM flap during the fluid-air exchange, which may result in surgical failure [8]. To improve the approach, we use a small amount of VISCOAT to surround the MH and cover the inverted ILM flap. This serves a dual purpose: acting as an adhesive and providing weight to maintain the flap during the fluid-gas exchange. Additionally, we administer VISCOAT into both the MH and the area above the MH that is symmetrical to the mirror image before injecting the ICG solution. This is done to minimize the potential toxicity of the ICG [15]. VISCOAT may be left in place without causing any harm to the retina. Consequently, in our cases, we did not observe any dislodged inverted ILM flaps during or after the surgery. Furthermore, the surgical closure rate for MHs was 98% in our study. MHs measuring less than 400 μm achieved 100% closure, whereas MHs ranging from 400 to 800 μm attained a closure rate of 98%. Very large MHs, exceeding 800 μm, achieved a closure rate of 91.3% in our case series. This closure rate, particularly for MH larger than 400 μm, is significantly higher than previously reported rates [16]. We believe that the modification of the viscoelastic-assisted inverted ILM (VILM) flap technique contributed to our high success rate, which is further supported by having a larger series of patients and by categorizing the study patients according to different sizes of MH.

The use of inverted flap procedures, including both temporal [10] and superior [17, 18] ILM flaps, has been shown to result in superior anatomical and functional recoveries compared to the traditional method of ILM peeling. Gliosis can occur as a result of ILM peeling, which involves Müller cell fragments. The gliosis may manifest on the surface of the ILM and within the retina when a section of the peeled ILM remains attached to the MH. Consequently, the ILM could potentially serve as a scaffold to promote tissue proliferation [19]. An Italian study involving 27 eyes with MH (> 400 μm) further compared the outcomes of the inverted ILM flap technique, with a single-layer ILM inverted over the MH, and a multiple layer ILM folded within the MH [20]. Both groups achieved a high anatomical success rate and significant visual improvement, except in the case of exceptionally large MHs (> 700 um), which closed more efficiently (0/2 vs. 2/2 cases) when employing the multilayer ILM. The primary distinction in our study lies in the use of a substantial single-layer inverted ILM flap to fill the MH, yielding promising clinical outcomes. Utilizing a bundle of multilayer ILM can be beneficial for facilitating anatomical restoration by effectively sealing and bridging the hole. Nevertheless, the inverted ILM acts as a framework for the rapid growth of glial cells within the MH [19], creating a conducive environment for photoreceptors to migrate closer to the fovea. Excessive insertion of ILM may lead to excessive fibrosis in the central macula, potentially hindering subsequent functional recovery. Our approach involved partially removing the inferior ILM in a half-circular manner to create a flap approximately the size of one disk diameter. This flap was then flipped to cover the MH from its upper edge, considering the force of gravity. As a result, the MH was treated by applying a large single-layer inverted ILM flap. We hypothesize that the presence of a single-layer ILM over the MH may facilitate a more organized and natural structure for the growth of glial cells, aiding in the closure of the MH without excessive fibrous tissue formation in the fovea.

Our cases demonstrated that 86.5% of the eyes showed a noticeable improvement in BCVA at 3 months and 6 months postoperatively. This finding aligns with a meta-analysis that concluded the inverted ILM flap technique is more effective than traditional ILM peeling in achieving better visual outcomes at three or more months after surgery for large MHs [14]. However, some meta-analyses have reported no difference in visual outcomes between the inverted ILM flap technique and traditional ILM peeling groups at 6-month follow-up, despite observing higher closure rates in the inverted ILM flap technique [21, 22]. The variation in results may be attributed to the relationship between baseline visual acuity (VA), the type of MH closure, and the final visual outcome. We found a correlation between BCVA improvement and baseline VA (r = 0.76, *p* = 0.03). Additionally, an impressive 91% of our patients with Type 1 closure exhibited a foveal configuration resembling either a U-shape or V-shape, resulting in a significant improvement in vision. Conversely, patients with Type 2 closure–characterized by the absence of the retinal pigment epithelium, photoreceptors, and external limiting membrane in the central area as observed through OCT–experienced poor visual outcomes during the follow-up period.

Several novel surgical techniques have been developed to address challenging MHs, such as the inverted ILM flap combined with an autologous blood clot technique, the neurosensory retinal flap, the perfluorocarbon liquid-assisted inverted ILM flap technique, the ILM transposition and tuck technique, and the use of a human amniotic membrane plug [16]. We utilized vitrectomy in combination with the VILM flap approach for treating MHs.

In conclusion, our case series demonstrated that the VILM flap technique is a significant surgical method with favorable success rates in treating large MHs [23]. Appropriate use of VISCOAT can effectively prevent dislocation or backward rotation of the ILM flap during fluid-gas exchange and mitigate the toxic effects of ICG staining on the retinal pigment epithelium. Implementing this procedure has the potential to enhance the success rate of the original operation while optimizing both the morphological and functional outcomes. We strongly endorse this straightforward and cost-effective technique for repairing macular holes.

## Supplementary Information

Below is the link to the electronic supplementary material.

Supplementary file1 (XSPF 1 KB)

## Data Availability

No datasets were generated or analysed during the current study.
